# Delineation of downstream signalling components during acrosome reaction mediated by heat solubilized human zona pellucida

**DOI:** 10.1186/1477-7827-8-7

**Published:** 2010-01-23

**Authors:** Beena Bhandari, Pankaj Bansal, Pankaj Talwar, Satish K Gupta

**Affiliations:** 1 Reproductive Cell Biology Laboratory, National Institute of Immunology, Aruna Asaf Ali Marg, New Delhi-110 067, India; 2 Assisted Reproduction Technology Centre, Army Hospital Research & Referral, Delhi Cantonment, Delhi-110 010, India

## Abstract

**Background:**

Human egg is enveloped by a glycoproteinaceous matrix, zona pellucida (ZP), responsible for binding of the human spermatozoa to the egg and induction of acrosomal exocytosis in the spermatozoon bound to ZP. In the present manuscript, attempts have been made to delineate the downstream signalling components employed by human ZP to induce acrosome reaction.

**Methods:**

Heat-solubilized human ZP (SIZP) was used to study the induction of acrosome reaction in capacitated human spermatozoa using tetramethylrhodamine isothiocyanate conjugated *Pisum sativum *agglutinin (TRITC-PSA) in absence or presence of various pharmacological inhibitors. In addition, intracellular calcium ([Ca2+]i) levels in sperm using Fluo-3 acetoxymethyl ester as fluorescent probe were also estimated in response to SIZP.

**Results:**

SIZP induces acrosomal exocytosis in capacitated human sperm in a dose dependent manner accompanied by an increase in [Ca2+]i. Human SIZP mediated induction of acrosome reaction depends on extracellular Ca2+ and involves activation of Gi protein-coupled receptor, tyrosine kinase, protein kinases A & C and phosphoinositide 3 (PI3)- kinase. In addition, T-type voltage operated calcium channels and GABA-A receptor associated chloride (Cl-) channels play an important role in SIZP mediated induction of acrosome reaction.

**Conclusions:**

Results described in the present study provide a comprehensive account of the various downstream signalling components associated with human ZP mediated acrosome reaction.

## Background

Zona pellucida (ZP), a glycoproteinaceous matrix that surrounds the mammalian oocyte, plays an important role in species-specific binding of the spermatozoon to the oocyte, induction of acrosomal exocytosis in the ZP-bound spermatozoa, avoidance of polyspermy and protection of the pre-implanted blastocyst. Human ZP matrix is composed of four glycoproteins designated as ZP1, ZP2, ZP3 and ZP4 whereas mouse ZP lacks ZP4 by virtue of it being a pseudogene. To accomplish fertilization, ZP mediated induction of acrosomal exocytosis is crucial that enables spermatozoa to penetrate the ZP matrix. In mouse, ZP3 is primarily responsible for induction of acrosome reaction [1,2] whereas in humans, ZP4 in addition to ZP3 contributes in induction of acrosome reaction [3-6]. Recent studies from our group suggest that in humans, ZP1 may also be involved in induction of acrosomal exocytosis (unpublished observations). It has also been proposed that a mechanosensory signal produced during zona penetration may also be required to initiate acrosome reaction [7].

At least, two different receptor mediated signalling pathways in sperm plasma membrane have been shown to be responsible for ZP-induced acrosomal exocytosis. One is a G_i _protein-coupled receptor that activates the Phospholipase C β1 (PLCβ1)-mediated signalling pathway and the other is a tyrosine kinase receptor coupled to PLCγ [6,8-10]. Activation of these pathways result in an increase of intracellular calcium ([Ca^2+^]_i_). The increase in [Ca^2+^]_i _and pH subsequently lead to fusion of sperm plasma membrane with Outer Acrosomal Membrane resulting in acrosome reaction and release of the acrosomal contents.

Studies done with the mouse ZP solubilized by either acid disaggregation or heat have shown to induce acrosome reaction and ability to increase [Ca^2+^]_i _which involves activation of G_i _protein-coupled receptor, T-type calcium channels and tyrosine kinase [11-13]. Incubation of capacitated human sperm with intact human zona or acid- disaggregated zonae led to a significant increase in acrosome reaction [14]. The acrosome reaction mediated by human ZP involves activation of G_i _protein-coupled receptor [15-17].

Keeping in view the differences in the composition of mouse *vs *human ZP matrix and the recent observations that in humans more than one zona protein may be involved in induction of acrosome reaction, in the present manuscript, we have delineated various downstream signalling components associated with human ZP mediated induction of acrosome reaction in human sperm employing various pharmacological inhibitors.

## Methods

### Isolation and solubilization of human zonae

In these investigations, unfertilized oocytes used were donated by patients from Assisted Reproduction Technology Centre, Army Hospital Research & Referral, New Delhi following project approval by the respective Institutional Human Ethical Committees and signed patient consent. The follicular fluid from women undergoing In Vitro Fertilization (IVF) treatment was aspirated under general anaesthesia and aseptic conditions. Oocyte-cumulus complex (OCC) were immediately separated under stereo zoom microscope (Zeiss, Baden-Wuerttenberg, Germany) and maintained in Universal IVF Medium (MediCult a/s, Mellehaven 12, Denmark) under liquid paraffin (MediCult a/s) and were inseminated with 0.1 × 10^6 ^motile sperm per OCC. Fertilization was confirmed after 17-24 hr by appearance of two pronuclei or second polar body. Those oocytes that failed to show the two pronuclei or the second polar body were further incubated for 12 hr and in absence of evidence of fertilization, they were stored in Embryo Freezing Medium (MediCult a/s) in liquid nitrogen until used in the present study. Prior to use, the oocytes were thawed, washed three times in 50 mM phosphate buffer (pH 7.4) containing 150 mM NaCl (PBS) and vigorously pipetted with small bore glass pipette to remove ZP from oocyte. The suspension was centrifuged at 1800 ×g for 15 minutes to pellet down ZP. The zonae were re-suspended in PBS and heat-solubilized at 70°C for 90 min. This preparation was designated as human SIZP.

### Induction of acrosome reaction by SIZP

All experiments using human spermatozoa were carried out under informed consent and following the clearance from the Institutional Bio-safety and Human Ethical Committee. Semen samples were collected from healthy donors after 3 days of sexual abstinence. Semen samples were assessed for volume, total sperm count, sperm morphology and sperm motility as per the WHO guidelines [18]. Semen samples showing sperm count of less than 20 million/ml or sperm motility less than 70% were not included in the present study. Semen samples from individual donors were processed separately and subjected to liquefaction at room temperature (RT) for 30 min. The motile sperm were isolated by two-step Percoll density gradient as described previously [19]. The sperm (10 × 10^6 ^cells/ml) were capacitated in Biggers-Whitten-Whittingham medium [20] supplemented with 2.6% BSA for 6 h at 37°C with 5% CO_2 _in humidified air in aliquots of 1 ml. Capacitated sperm (1 × 10^6 ^in BWW + 0.3% BSA) were incubated at 37°C with 5% CO_2 _in humidified air for 1 hr in presence of SIZP in a total reaction volume of 100 μl. For measurement of spontaneous induction of acrosome reaction, sperm were also incubated with BWW + 0.3% BSA alone. Calcium ionophore (10 μM, A23187; Sigma-Aldrich Inc., St. Louis, MO, USA) served as a positive control in all the experiments. Post-incubation, the sperm were washed with 50 mM PBS pH 7.4, assessed for sperm viability by one step eosin-nigrosin staining method [21] and 20 μl aliquots were spotted on poly-L-Lysine coated slides (Sigma-Aldrich Inc.) in duplicates. The spots were air-dried, fixed in chilled methanol for 30 seconds and stained with 5 μg/ml tetramethylrhodamine isothiocyanate conjugated *Pisum sativum *agglutinin (TRITC-PSA; Vector Laboratories Inc., Burlingame, CA, USA) for 30 min at RT. Any spermatozoa that demonstrated complete loss of TRITC-PSA staining in the acrosome or revealed staining at the equatorial region was classified as acrosome-reacted. Sperm showing TRITC fluorescence in the acrosomal region of the head were classified as acrosome intact. All the slides were read "blind" with coded samples under Nikon Eclipse 80 *i *epifluorescence microscope (Nikon, Chiyoda-ku, Tokyo, Japan) using an oil immersion objective. Two hundred sperm were scored for every spot and the percentage of acrosome reaction was calculated by dividing the number of acrosome-reacted sperm by the total number of sperm counted and multiplied by hundred. Induction of acrosome reaction at an optimized dose of SIZP was evaluated using semen samples from six different donors.

### Intracellular calcium estimation

Changes in [Ca^2+^]_i _were analyzed with the fluorescent probe Fluo-3 acetoxymethyl (AM) ester (Molecular Probes, Eugene, OR, USA). Capacitated sperm (10 × 10^6^) were loaded with 2 μM fluo-3 AM containing 1 μM pluronic acid F-127 (Molecular Probes; for proper dispersal of dye) for 1 hr at 37°C with 5% CO_2 _in air. Labelled sperm were then kept for half an hour at 37°C with 5% CO_2 _in air for de-esterification of dye. Labelling and de-esterification of Fluo-3 AM in capacitated sperm was performed in BWW medium whereas assay was performed in BWW medium supplemented with 0.3% BSA. Capacitated sperm (1 × 10^6^/well) were added in 96 well black plates (BMG Technologies, Offenburg, Germany). The baseline fluorescence measurements were performed at an excitation wavelength of 480 nm and an emission of 520 nm for ~200 sec followed by addition of SIZP and continued fluorescence measurements for next ~10 minutes. The [Ca^2+^]_i _was calculated by using the Grynkiewicz equation [Ca^2+^]_i _= K_d _(F-F_min_)/(F_max_-F), where K_d _is the dissociation constant of the Ca^2+^-fluo-3 complex (390 nM), and F represents the fluorescence intensity of the cells [22]. F_max _represents the maximum fluorescence (obtained by treating cells with 1% Triton-X) and F_min _corresponds to the minimum fluorescence (obtained in the presence of 8 mM EGTA). Ca^2+ ^levels [nM] have been presented as the change in intracellular calcium, Δ [Ca^2+^]_i _by calculating difference between peak [Ca^2+^]_i _and resting [Ca^2+^]_i _before stimulation. Resting [Ca^2+^]_i _represent the average of sperm [Ca^2+^]_i _for 200 sec preceding SIZP addition. All measurements were carried out in a Fluostar Optima Spectrofluorimeter (BMG Technologies).

### Delineation of voltage operated calcium channels (VOCCs) associated with SIZP mediated release of [Ca^2+^]_i _and acrosome reaction

To delineate the involvement of different type of Voltage Operated Calcium Channels (VOCCs) during SIZP mediated induction of acrosome reaction, 1 × 10^6 ^capacitated sperm were pre-treated with Pimozide (10 and 20 μM) or Mibefradil (5 and 10 μM) as T-Type Ca^2+ ^channel blocker (CCB); Verapamil (10 and 20 μM) or Nifedipine (10 and 20 μM) as L-Type CCB; for 10 min at 37°C with 5% CO_2 _in humidified air prior to the addition of SIZP. The concentrations of the various inhibitors employed in these experiments were based on previously published studies [6,23-26] and inhibitors were procured from Sigma-Aldrich Inc. In addition, effect of prior treatment of capacitated human sperm with Pimozide and Verapamil on the levels of [Ca^2+^]_i _in response to SIZP were also determined by fluorimetric assay as described above.

### Delineation of downstream signalling components associated with SIZP mediated induction of acrosomal exocytosis

To understand the mechanism of action of human SIZP mediated acrosome reaction, 1 × 10^6 ^capacitated spermatozoa were pre-treated with various pharmacological inhibitors such as Picrotoxin (50 and 100 μM) - GABA_A _receptor antagonist [27]; Pertussis toxin (PTX, 0.1 μg/ml) - G_i _protein-coupled receptor pathway inhibitor [6,16,28,29]; Herbimycin-A (10 and 15 μM) - tyrosine kinase inhibitor [6,30]; Chelerythrine chloride (2 and 3 μM) - protein kinase C inhibitor [6,30]; Wortmannin-A (50 and 100 nM) - phosphoinositide 3-kinase (PI-3 kinase) inhibitor [31]; H-89 (20 μM) - cAMP dependent protein kinase A inhibitor [6,30] for 1 hr (except Picrotoxin, pre-treated for 10 min) at 37°C with 5% CO_2 _in humidified air prior to the addition of human SIZP. To study the relevance of extra-cellular Ca^2+^, capacitated sperm were either pre-incubated for 10 min with 8 mM EGTA or added at the same time as SIZP. All the above inhibitors were procured from Sigma-Aldrich Inc.

### Statistical analysis

The results pertaining to SIZP mediated induction of acrosome reaction are presented as mean ± SEM and statistical analysis was done by comparing the means of the medium control (BWW + 0.3% BSA)/vehicle control and experimental sets or within two experimental groups by using paired Student's t-test/Wilcoxon signed rank test. A value of p < 0.05 was considered to be statistically significant.

## Results

### SIZP induces acrosomal exocytosis in capacitated human sperm in a dose dependent manner

A significant increase in the induction of acrosomal exocytosis of capacitated human sperm was observed with a concomitant increase in number of zonae equivalent (SIZP) used per reaction, as compared to PBS (solvent used in preparation of SIZP; Table 1). As low as ~1 zona equivalent was able to induce statistical significant induction of acrosome reaction in capacitated human sperm. However, no further increase in acrosomal exocytosis was observed with SIZP preparation from more than ~5 zonae per reaction. Subsequently, 5 zonae equivalent SIZP was used in all experiments. Capacitated sperm prepared from 6 different donors on incubation with optimized concentration of human SIZP showed a significant (p = 0.007) increase in induction of acrosome reaction (Table 1).

**Table 1:** Human SIZP mediated induction of acrosome reaction in capacitated human sperm

Treatment	Percent induction of acrosomal exocytosis (Mean ± SEM)	Statistical significance
***Experiment I*^a^**		
PBS Control	13.7 ± 1.4	
SIZP (~1 zona)	26.3 ± 1.4	p = 0.003^c^
SIZP (~2 zonae)	29.4 ± 0.4	p = 0.009^c^
SIZP (~5 zonae)	39.7 ± 1.7	p = 0.0003^c^
SIZP (~10 zonae)	36.4 ± 0.9	p = 0.0009^c^
***Experiment II*^b^**		
PBS control	14.7 ± 1.3	
PBS + SIZP (~5 zonae)	36.1 ± 3.1	p = 0.007^c^
Calcium ionophore (10 μM)	56.1 ± 4.5	p = 0.0001^c^

^a^SIZP mediated acrosome reaction was studied by employing semen samples from three different donors

^b^SIZP mediated acrosome reaction was studied by employing semen samples from six different donors

^c^Values are statistically significant as compared to respective PBS control

### T-type VOCCs are responsible for SIZP mediated induction of acrosome reaction subsequent to an initial increase in [Ca^2+^]_i_

**Figure 1:**
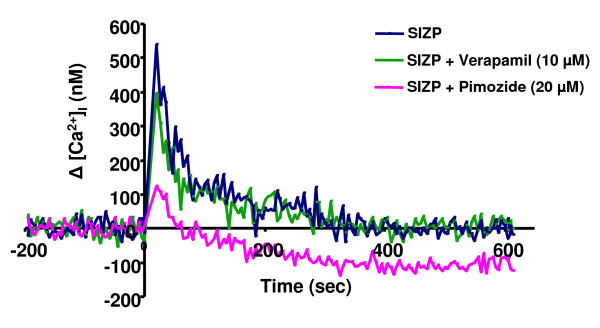
**SIZP mediated intracellular calcium release profile in capacitated human sperm in presence or absence of pharmacological inhibitors of L- and T-type VOCCs**. Fluo-3/AM labelled capacitated human sperm (1 × 10^6^) were pre-incubated with either L- type VOCC inhibitor, Verapamil (10 μM) or T-type VOCC inhibitor, pimozide (20 μM), for 10 minutes and then exposed to SIZP (~5 zonae) at 0 seconds. Changes in intracellular calcium levels (nM; y-axis) have been plotted as a function of time (seconds; x-axis) for ~10 min. Blue line represents SIZP mediated calcium increase profile whereas pink and green lines represent SIZP mediated calcium release profile after prior treatment of labelled sperm with Pimozide and Verapamil respectively. Values are presented as Δ [Ca^2+^]_i _obtained by subtracting the respective mean resting [Ca^2+^]_i _values preceding SIZP addition from the peak [Ca^2+^]_i_.

An increase in [Ca^2+^]_i_, after coming in contact with ZP, is a prerequisite for induction of acrosomal exocytosis in mammalian sperm [32]. In the present study, SIZP was also able to elicit an increase in [Ca^2+^]_i _after incubating with fluo-3/AM labelled capacitated human sperm (Fig. 1). To decipher the type of VOCCs playing an important role in human SIZP mediated increase in initial [Ca^2+^]_i _surge as well as subsequent induction of acrosome reaction, pharmacological inhibitors for L- and T- type VOCCs were employed. Prior-incubation (10 min) of fluo-3/AM labelled capacitated human sperm with Pimozide (20 μM; T- type VOCC inhibitor) inhibited the SIZP mediated initial increase in [Ca^2+^]_i_surge, whereas Verapamil (10 μM; L- type VOCC inhibitor) failed to do so (Fig. 1).

**Figure 2:**
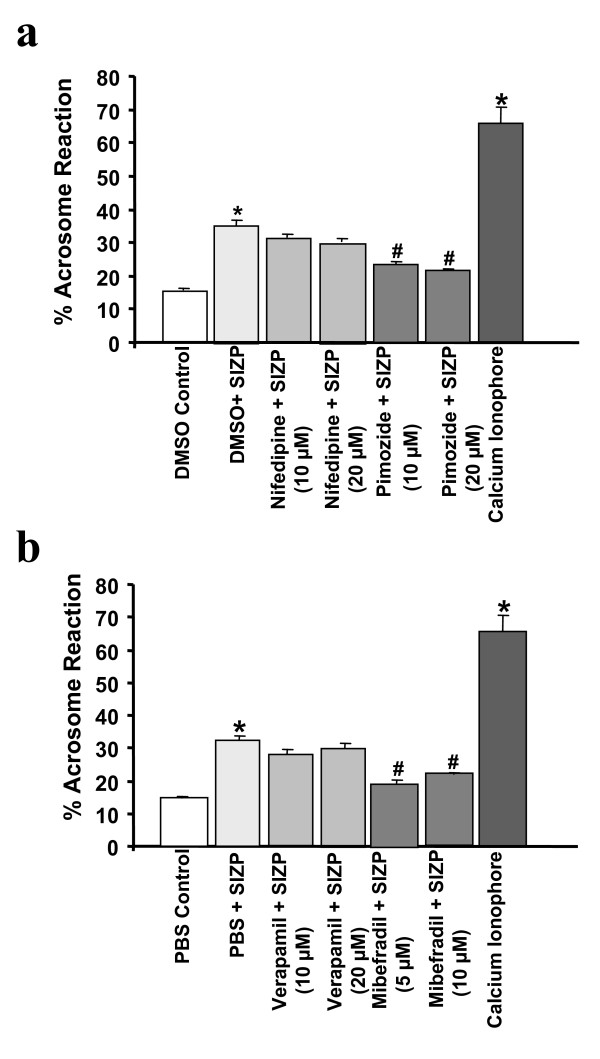
**SIZP mediated induction of acrosome reaction in capacitated human sperm in presence or absence of pharmacological inhibitors of L- and T-type VOCCs**. Capacitated human sperm (1 × 10^6^) pre-incubated for 10 min at 37°C in presence of 5% CO_2_, with L-type VOCC inhibitors (Nifedipine, Verapamil), T-type VOCC inhibitors (Pimozide, Mibefradil) or respective vehicle controls (Panels 2a and 2b respectively) were subsequently incubated with SIZP (~5 zonae). After 60 min incubation at 37°C in presence of 5% CO_2_, sperm samples were analyzed for their acrosomal status by TRITC-PSA staining as described in *Methods*. Y-axis represents percent induction of acrosomal exocytosis calculated by dividing the number of acrosome-reacted sperm by total number of sperm counted and multiplied by 100. Values are Mean ± SEM of six different experiments using semen samples from at least six different male donors. Calcium ionophore A23187 (10 μM) was used as a positive control in the experiment. Asterisk (*) represents p < 0.05 with respect to the solvent control and hash (#), p < 0.05 with respect to SIZP mediated induction of acrosome reaction.

To further assess the importance of these VOCCs, induction of acrosome reaction in capacitated human sperm was quantitated after incubation with SIZP in presence or absence of pharmacological inhibitors of L- or T-type specific VOCC. Pre-incubation of capacitated human sperm with T-type VOCC inhibitors, Pimozide (10 and 20 μM) or Mibefradil (5 and 10 μM) significantly reduced the SIZP mediated induction of acrosome reaction whereas L-type VOCC inhibitors, Nifedipine (10 and 20 μM) or Verapamil (10 and 20 μM) failed to inhibit the same (Fig. 2a, b).

Pre-incubation of capacitated human spermatozoa with EGTA (8 mM) for 10 min was able to significantly (p = 0.001) inhibit SIZP mediated induction of acrosome reaction (Table 2). Further, capacitated sperm after re-suspension in the EGTA medium were immediately exposed to human SIZP. Under these experimental conditions, 16 ± 0.6% sperm showed acrosome reaction in presence of EGTA as compared to 36.1 ± 1.0% in its absence (p = 0.0001), suggesting the role of extra-cellular calcium in SIZP mediated induction of acrosomal exocytosis in human sperm. Addition of 8 mM EGTA led to negligible levels of free calcium in the reaction medium as analyzed by Maxchelator programme [33].

**Table 2:** Human SIZP mediated induction of acrosome reaction in capacitated human sperm in presence or absence of various pharmacological inhibitors.

Treatment	Percent induction of acrosomal exocytosis (Mean ± SEM)	Statistical significance
***Experiment I*^a^**		
PBS control	14.5 ± 0.5	
PBS + SIZP	31.1 ± 1.2	p = 0.04*^b^
EGTA (8 mM) + SIZP	16.2 ± 0.9	p = 0.001*^c^
Pertussis toxin (0.1 μg/ml) + SIZP	21.1 ± 0.8	p = 0.02*^c^
H-89 (20 μM) + SIZP	19.8 ± 0.9	p = 0.02*^c^
***Experiment II*^a^**		
Alcohol control	14.5 ± 0.7	
Alcohol + SIZP	28.9 ± 1.0	p = 0.0004*^b^
Picrotoxin (50 μM) + SIZP	23.4 ± 0.5	p = 0.005*^c^
Picrotoxin (100 μM) + SIZP	21.3 ± 0.5	p = 0.0007*^c^
***Experiment III*^a^**		
DMSO Control	15.7 ± 1.4	
DMSO + SIZP	36.0 ± 1.4	p = 0.0001*^b^
Chelerythrine chloride (2 μM) + SIZP	21.5 ± 1.4	p = 0.006*^c^
Chelerythrine chloride (3 μM) + SIZP	21.2 ± 2.4	p = 0.007*^c^
Wortmannin A (50 nM) + SIZP	22.3 ± 0.6	p = 0.0001*^c^
Wortmannin A (100 nM) + SIZP	25.0 ± 1.7	p = 0.008*^c^
Herbimycin A (10 μM) + SIZP	21.0 ± 1.2	p = 0.012*^c^
Herbimycin A (15 μM) + SIZP	19.1 ± 0.9	p = 0.02*^c^

^a^Capacitated human sperm (1 × 10^6^) pre-treated with or without respective inhibitor were incubated with SIZP (5 zonae) for 60 min

^b^Values are statistically significant as compared to PBS control

^c^Values are statistically significant as compared to SIZP

### SIZP mediated induction of acrosome reaction involves activation of Gi pathway, PKA, PKC, PI3 Kinase, Tyrosine Kinase and GABA_A_ receptor associated Cl^-^ channels

SIZP mediated induction of acrosomal exocytosis (31.1 ± 1.2%) was inhibited in presence of Pertussis toxin (0.1 μg/ml) to a statistically significant extent (21.1 ± 0.8%; p = 0.02; Table 2). Acrosomal exocytosis mediated by SIZP was also inhibited by prior incubation of capacitated human sperm with either 50 or 100 μM GABA_A _receptor antagonist, Picrotoxin (from 28.9 ± 1.0% to 23.4 ± 0.5% & 21.3 ± 0.5% respectively) and cAMP dependent protein kinase A inhibitor, H-89 (from 31.1 ± 1.2% to 19.8 ± 0.9%) (Table 2). In addition, pre-incubation of capacitated human sperm with inhibitors of other kinases such as, PKC (Chelerythrine chloride), PI3-kinase (Wortmannin A) and tyrosine kinase (Herbimycin A) also led to inhibition of SIZP mediated acrosomal exocytosis (Table 2).

## Discussion

The acrosome reaction, an exocytotic process, is essential for fertilization in all sperm species possessing an acrosome. In response to the physiological egg inducer or to an appropriate pharmacological stimulus, the outer acrosome membrane and the overlying plasma membrane fuse and vesiculate, leading to exposure of the acrosomal contents, inner acrosomal membrane and modified plasma membrane to the extracellular medium [34]. The ZP has been implicated as the primary physiological inducer responsible for acrosomal exocytosis of the egg bound spermatozoa [35,36]. The molecular basis of induction of acrosome reaction has been investigated in detail employing mouse ZP [37,38]. On the other hand, there are few studies pertaining to the role of human ZP mediated acrosome reaction primarily due to limited availability of human eggs due to ethical considerations [14-16].

The human SIZP prepared by heat solubilization induced acrosomal exocytosis in a dose dependent fashion which is in agreement with previous studies wherein acid-disaggregated human ZP was employed [14]. The observed extent of acrosome reaction by human SIZP is within the range described by other investigators [14,17,29,39]. The kinetics and extent of acrosome reaction mediated by solubilized zona differ from species to species. One of the possible explanations for SIZP mediated lower acrosome reaction observed in humans may be due to lesser degree of capacitation achieved by human sperm using *in vitro *conditions as compared to that achieved *in vivo*. Further, mechanosensory signals produced during penetration of spermatozoa through zona matrix may also contribute to higher levels of acrosome reaction [7].

Calcium is an important second messenger in spermatozoa of various species including mammals and is required for acrosomal exocytosis. In the present studies, incubation of the capacitated human sperm with SIZP resulted in transient calcium peak. VOCCs are important mediators of early intracellular calcium influx which are activated on membrane potential changes following agonist binding. In this manuscript, we have identified type of VOCCs responsible for the early intracellular calcium influx as well as their role in acrosomal exocytosis mediated by SIZP in human sperm. Prior treatment with T-type VOCC inhibitor, Pimozide abolished the early [Ca^2+^]_i _peak whereas L-type inhibitor Verapamil failed to do so. Role of T-type VOCCs was further reinforced by inhibition of acrosome reaction mediated by human SIZP in presence of two different T-type VOCCs inhibitors (Pimozide and Mibefradil). Further, chelating the extracellular calcium by EGTA also led to inhibition of SIZP mediated acrosome reaction. In contrast to T-type VOCCs inhibitors, L-type VOCCs inhibitors (Nifedipine and Verapamil) failed to inhibit SIZP mediated acrosome reaction. Patrat *et al*., [26] has shown that solubilized zona prepared from unfertilized and fertilized human eggs induces acrosome reaction and increase in [Ca^2+^]_i _is mediated by T-type VOCC. However, the ability of SIZP prepared from fertilized eggs to induce acrosome reaction needs further investigation.

Besides, being an important inhibitory neurotransmitter in the central nervous system, γ Aminobutyric acid (GABA) also operates in the human genital tract. γ aminobutyric acid receptors and the GABA uptake system are present in both male and female genital tract. A specific binding and transport system is present on the plasma membrane of the human spermatozoon [40]. GABA also induces acrosome reaction in human sperm [40-42]. Out of two classified GABA receptor subtypes GABA_A _and GABA_B_, GABA_A _receptor is a plasma membrane multi-subunit receptor complex linked to the chloride channel whose activation results in the opening of the chloride channel. Progesterone and its metabolites potentiate the effects of GABA on this receptor [43]. Picrotoxin - a GABA_A _receptor inhibitor, inhibits progesterone as well as recombinant human ZP3 fragment (214-348 aa) mediated acrosome reaction [27,44,45]. Studies presented in this manuscript suggest that in humans, ZP mediated induction of acrosome reaction is also inhibited by inhibitor of GABA_A _receptor.

Heat solubilized human ZP mediated acrosome reaction involves activation of G_i _protein- coupled receptor pathway which is in concordance with previous reports [15-17,29]. Recent studies employing either recombinant human zona proteins or immunoaffinity purified native human zona proteins revealed that ZP3 mediated induction of acrosome reaction involves activation of Pertussis toxin sensitive G_i _protein-coupled receptor pathway whereas ZP4 mediated induction of acrosome reaction is not dependent on the activation of G_i _protein-coupled receptor pathway [3,6]. This may explain partial but statistically significant inhibition of acrosome reaction by human SIZP in presence of Pertussis toxin (Table 2).

One major component of signal transduction cascade downstream to G_i _protein is adenylate cyclase that generates second messenger cAMP upon its activation. cAMP in turn binds and activates protein kinase A in addition to other kinases. In humans, pharmacological inhibition of cAMP dependent PKA by KT5720 has been shown to reduce SIZP induced acrosome reaction [46]. Native purified human ZP4 but not ZP3, mediated induction of acrosome reaction has been shown to be inhibited in capacitated human sperm following pre-treatment with H-89, pharmacological inhibitor of PKA [6]. Our findings with human SIZP which contain all four zona proteins showed a significant inhibition (p < 0.05) in induction of acrosome reaction in presence of H89; thereby suggesting that human ZP mediated acrosome reaction involves other zona proteins in addition to ZP4.

Various other kinases are also involved in ZP mediated acrosome reaction either through direct or indirect activation of downstream effector molecules in the signalling cascade. An important role of protein kinase C in human ZP induced acrosome reaction has been suggested employing human oocytes, where PKC activator, Phorbol 12-myristate 13-acetate (PMA), showed enhanced human ZP induced acrosome reaction and PKC inhibitor, staurosporine, decreased extent of acrosome reaction [47]. In humans, SIZP induced acrosome reaction has also been shown to be inhibited by PKC inhibitor, Calphostin [46]. Native purified human ZP3 and ZP4 mediated acrosome reaction also showed an inhibition in acrosome reaction following PKC inhibitor, chelerythrine chloride pre-treatment [6]. Our findings with solubilized zona also highlight the role of PKC in zona induced acrosome reaction. The importance of both PKA and PKC pathways is further emphasised during fertilization by the observations of enhanced sperm-ZP binding in presence of PKA (dbcAMP) and PKC (PMA) activators [48].

Recent studies in murine system implicate important role of PI 3-kinase in ZP induced acrosome reaction [49]. Treatment of capacitated mouse sperm with ZP3 stimulates production of phosphatidylinositol-(3,4,5)-triphosphate and which in turn activates protein kinases, Akt (Protein kinase B) and PKCζ, which function as downstream effectors of phosphoinositide signalling. Capacitated mouse sperm pre-treated with two different pharmacological inhibitors of PI 3-kinase, Wortmannin or LY294002, before exposure to either a soluble extract of zonae or with purified ZP3 resulted in 90% inhibition in acrosome reaction [49]. In human sperm the relevance of PI 3-kinase has been demonstrated in mannose-bovine serum albumin (mannose-BSA) mediated acrosome reaction. Wortmannin was shown to inhibit the mannose-BSA mediated acrosomal exocytosis but not that induced by calcium ionophore, A23187 or by progesterone [31]. In this manuscript, for the first time, we have shown the role of PI 3-kinase in human SIZP mediated acrosome reaction. Selective inhibitor of PI 3-kinase, Wortmannin, significantly inhibited the acrosomal exocytosis induced by human SIZP. Further for the first time, we have shown that tyrosine kinase has an important role in SIZP mediated induction of acrosome reaction (Table 2).

In conclusion, an attempt has been made to delineate various signalling components that are involved in human ZP mediated acrosome reaction. Better understanding of the signalling pathways associated with ZP mediated induction of acrosome reaction may help in optimizing protocols aiming to increase *in vitro *fertilization rate or development of novel contraceptives to block fertilization.

## Competing interests

The authors declare that they have no competing interests.

## Authors' contributions

BB performed the experiments. PB helped with the experiments. PT helped with oocyte collection from IVF patients. SKG designed the study and finalized writing of the manuscript. All the authors have read and approved the final manuscript.
